# On the complexity of IgE: The role of structural flexibility and glycosylation for binding its receptors

**DOI:** 10.3389/falgy.2023.1117611

**Published:** 2023-03-28

**Authors:** Kevin Plattner, Martin F. Bachmann, Monique Vogel

**Affiliations:** ^1^ Department of Immunology, University Clinic for Rheumatology and Immunology, University of Bern, Bern, Switzerland; ^2^ Department of Biomedical Research Bern (DBMR), University of Bern, Bern, Switzerland; ^3^ Nuffield Department of Medicine, The Jenner Institute, University of Oxford, Oxford, United Kingdom

**Keywords:** IgE, IgE-immune complexes, Fc epsilon receptors, galectins, Fc gamma receptors (Fc*γ*R), glycosylation

## Abstract

It is well established that immunoglobulin E (IgE) plays a crucial role in atopy by binding to two types of Fcε receptors (FcεRI and FcεRII, also known as CD23). The cross-linking of FcεRI-bound IgE on effector cells, such as basophils and mast cells, initiates the allergic response. Conversely, the binding of IgE to CD23 modulates IgE serum levels and antigen presentation. In addition to binding to FcεRs, IgE can also interact with other receptors, such as certain galectins and, in mice, some FcγRs. The binding strength of IgE to its receptors is affected by its valency and glycosylation. While FcεRI shows reduced binding to IgE immune complexes (IgE-ICs), the binding to CD23 is enhanced. There is no evidence that galectins bind IgE-ICs. On the other hand, IgE glycosylation plays a crucial role in the binding to FcεRI and galectins, whereas the binding to CD23 seems to be independent of glycosylation. In this review, we will focus on receptors that bind to IgE and examine how the glycosylation and complexation of IgE impact their binding.

## Introduction

Immunoglobulin E (IgE) is a crucial molecule in the development of allergic disorders. It interacts with various receptors, with the FcεRI receptor being the most important and well-studied of these. Free IgE preferentially binds to FcεRI due to its high affinity for this receptor. The cross-linking of FcεRI by IgE and allergen triggers intracellular signaling, leading to the degranulation of effector cells such as basophils and mast cells. The mediators released in this process are responsible for the characteristic symptoms of allergies.

The second IgE receptor is CD23 (FcεRII), whose function has long been overlooked as important in the field of allergy. This is because CD23 binds IgE with lower affinity and is involved in different immunological processes (1, 2). However, recent research has demonstrated that CD23 is a crucial IgE receptor, offering a non-inflammatory pathway for IgE, regulating serum IgE levels, and playing a role in antigen presentation (3–10).

Galectin 3 and 9 are also receptors for IgE, and both are expressed in humans and mice (11–14). Beside their function as IgE receptors, little is known about the specific roles of Galectin 3 and 9 due to their involvement in many complex and diverse immunological processes (15–20). As receptors for IgE, they have the potential to exhibit both inflammatory and anti-inflammatory properties in mice and humans (14, 21–25).

So far, few studies have investigated the binding of IgE to Fc*γ* receptors. However, recent research performed in mice suggests that FcγII, FcγIII, and FcγIV, which correspond to FcεRI (αγγ) in humans, only bind IgE in a complexed form, such as when it is bound to an allergen or IgG (26, 27). Furthermore, the glycosylation of IgE has been shown to play a significant role in its binding to receptors, highlighting the importance of understanding the complexity of IgE's interaction with its receptors (28–30). Although still many questions remain unanswered about the functions of IgE receptors, this review aims to provide an overview of current knowledge on the topic and inspire further research.

## Structure, flexibility, and glycosylation of IgE

IgE, like all other antibodies, is composed of two identical light and heavy chains, each consisting of a variable (V) region and a constant (C) region. The C region of the IgE heavy chain contains four Ig domains (Cε1-Cε4) and one V domain (VH). The C region allows for interaction with its two main receptors, FcεRI and FcεRII (CD23). Compared to IgG, the IgE-Fc has an additional pair of domains (Cε2), and the Cε3-Cε4 domains of IgE are most homologous to the Cγ2-Cγ3 domains of IgG-Fc (31).

IgE is unique because it lacks a hinge region, and its flexible constant region can adopt either an open or closed conformation (31). The closed form has a more acute angle between the Cε3 and Cε4 domains, resulting in closer proximity of the Cε3 domains. Interestingly, the region responsible for this flexibility is located at the end of the Cε3 domain, near the Cε3-Cε4 linker. These different conformations have implications for binding to central IgE receptors, with soluble IgE or CD23-bound IgE in the closed form whereas IgE bound to FcεRI has an open form. This means that IgE cannot simultaneously bind both CD23 and FcεRI receptors (32).

Furthermore, IgE is the most glycosylated immunoglobulin, with about 12% of its molecular weight resulting from glycosylation (33), containing only N-glycans (33). N-glycans are a class of sugars that possess a common core structure, consisting of a sequence of two Acetylglucosamine (GlcNAc) attached to the amino acid, followed by three mannose residues. Their glycans based on this core can be classified into three categories: oligomannose (34), which features only mannose residues attached to the core structure; the complex type (35), which has “antennae” branches initiated by N-acetylglucosaminyltransferases (GlcNAcTs) that are attached to the core; and the hybrid type (36), which possesses mannose residues attached to one mannose arm of the core and one or two antennae on the other mannose arm.

Seven asparagine N-linked glycosylation sites for human IgE (37) and nine for murine IgE (30) are distributed across the constant domains. Most of them are complex glycan structures, consisting of different glycans, such as fucose, GlcNAc, mannose, galactose, and sialic acid. One site, at asparagine (N) 394 on human IgE and at N384 on mouse IgE, has an oligomannose structure that is conserved (30, 38–40). This oligomannose structure in IgE has 2–9 mannose residues attached to the core, with Man5GlcNAc2 being the primary form. The impact of glycosylation on the biological activity of IgE and how it differs in disease states are not well understood. A recent study performed with peanut-allergic and non-atopic individuals found that atopic individuals had increased numbers of sialic acid residues per IgE molecule, while healthy individuals had more biGlcNAc and terminal galactose residues (41).

## Modulating the FcεRI: role of IgE glycosylation and conformation

IgE antibodies bind to FcεRI on the surface of mast cells and basophils, which in turn leads to the release of inflammatory mediators such as histamine, prostaglandins, and leukotrienes upon cross-linking of cell-bound IgE with allergens. This process can result in a range of allergic symptoms, including the most severe form of allergic reaction, anaphylaxis.

In mice, FcεRI is expressed only as a tetrameric structure, consisting of the IgE-binding α-chain, two γ-chains responsible for signaling, and the β-chain which amplifies signaling of FcεRI (42, 43).

In contrast, in humans, FcεRI can also exist as a trimeric structure, lacking the β-chain, and is expressed on antigen-presenting cells such as dendritic cells (44), monocytes (45), and Langerhans cells (46, 47). These cells utilize the trimeric isoform of FcεRI for the uptake of antigens *via* IgE, which is then transported to endo/lysosomal compartments for loading and presentation on MHC class II molecules (44, 48, 49).

As a result of IgE high affinity for FcεRI (10^−10^ M) most IgE remains bound to the receptor on the cell surface for several weeks, leaving only a small amount of free, short-lived IgE in the plasma (50). The expression level of FcεRI is closely linked to the serum level of IgE in humans and mice (51, 52). In vitro studies have shown the ability of IgE to upregulate FcεRI on human basophils (51). Likewise, a reduction in the level of IgE in human serum results in a corresponding decrease in the amount of FcεRI expressed on cell surfaces (53, 54).

Additionally, studies have shown that the glycosylation of IgE plays a role in the strength of allergic reactions. Shade et al. found that individuals with peanut allergies have higher levels of sialic acid per IgE than non-allergic individuals (41). Removal of sialic acid from IgE attenuates the allergic reaction, while adding sialic acid can increase the allergic response in mice and humans. It is important to note that sialic acid did not affect the binding of IgE to FcεRI or of the allergen to receptor-bound IgE. Instead, the removal of sialic acid was reported to decrease phosphorylation of Syk (41). This suggests that IgE glycosylation plays a role in the strength of effector cell activation and the severity of allergic symptoms. The same group also showed that the evolutionarily conserved oligomannose is mandatory for IgE to bind to FcεRI. It appeared that the glycan is not directly involved in the binding but its removal leads to a slight change in the secondary structure, which probably explains the altered function of IgE (30). Similar changes in glycosylation also affect the function of IgG (55).

We have previously shown that complexed IgE (IgE-IC, i.e., bound to its specific allergen) decreases the ability of IgE to bind to FcεRI. At the present time the reason for this is not yet understood (3). One possible explanation is that the binding of allergen to IgE affects its conformation, limiting its flexibility and preventing it from adopting the open form required for binding to FcεRI (31). This phenomenon could represent a kind of negative feedback. In this way, IgE, which preferentially forms immune complexes when many allergens are present, can prevent the sensitization of new basophils. Likewise, IgE-allergen ICs are eliminated from the serum by increased binding to CD23 and cannot sensitize effector cells as they fail to bind to FcεRI (3). Therefore, removing the oligomannose of IgE and forming IgE-ICs may have a similar impact on IgE's conformation and thereby the ability to bind to FcεRI. However, further research is needed to analyze this hypothesis.

In addition to the membrane-bound form of FcεRI, a soluble version of FcεRI exists with a molecular weight of 40 kD instead of 60 kD for the full length protein. This is explained by the lack of the transmembrane and cytosolic domains (56, 57). It contains an IgE binding site and can be found in serum as a free form or as a complex with IgE (56). The production of sFcεRI *in vivo* has not yet been characterized, but *in vitro* data with human cells shows that it can be generated after IgE-mediated cross-linking of the trimeric form of surface-expressed FcεRI (56). It is unclear whether activation of tetrameric FcεRI induces the release of sFcεRI and if sFcεRI exists in mice. sFcεRI has several potential binding partners, including IgE, IgE-IC, and membrane IgE (mIgE) expressed by B-cells. When sFcεRI forms a complex with free IgE or IgE in complex with antigen, it could inhibit IgE binding to other FcεRI receptors on effector cells (56). This can prevent IgE-mediated cell activation, which can be beneficial for blunting the acute phase of an allergic response. However, if larger sFcεRI-IgE-allergen complexes interact with surface-expressed FcεRI, they could activate cells *via* FcεRI-crosslinking and exacerbate immediate type allergic responses (58). When sFcεRI block IgE from binding to antigen presenting cells such as dendritic cells IgE-mediated antigen presentation may be downregulated. This could affect the sensitization phase and Th2-type immune responses of chronic allergic reactions (59). Additionally, if sFcεRI is internalized as part of a sFcεRI-IgE-antigen complex by antigen-presenting cells, it could present FcεRI alpha-chain as an exogenous antigen and generate autoantibodies against FcεRI (60, 61).

Another potential mechanism for the development of anti-FcεRI antibodies is through the formation of anti-IgE-IgG-IgE-sFcεR complexes. In this scenario, FcγR on APCs could bind to the complexes, presenting the alpha-chain as an antigen and resulting in the generation of autoantibodies against FcεRI.

## FcεRII (CD23): a C-type lectin receptor

CD23 is not a typical Fc receptor since it does not belong to the Ig superfamily but to the C-type lectin family. According to a recent study, CD23 from mice and cows binds carbohydrates in a Ca^2+^-dependent manner whereas human CD23 does not (62). It is composed of three monomers in its membrane-bound state. Each monomer of CD23 is composed of a c-type lectin-like domain (CTLD) globular region at the C-terminus, connected to a hydrophobic membrane-spanning region by an α-helical coiled-coil stalk. The monomer also contains a short cytoplasmic domain at the N-terminus (63, 64).

A single CD23 domain binds IgE with a relatively low affinity of K_D_ = 10^−6^ M (1, 2), however by forming trimers the avidity effect can enhance the binding strength to IgE up to a K_D_ of 10^−8^ M (2, 65, 66).

In humans, CD23 is expressed on a variety of cells including B cells (67, 68), T cells (69, 70), monocytes (71–73), follicular dendritic cells (74), intestinal epithelial cells (75, 76), bone marrow stromal cells (77), and respiratory epithelial cells (78). This protein plays an important role in a wide range of immune functions, including the regulation of IgE synthesis, transport of IgE-immune complexes, antigen presentation, receptor-mediated endocytosis, cell survival, and cytokine release (68, 72, 73, 75, 76, 78–80).

In contrast to humans, the expression of CD23 in mice is restricted to B cells (81), macrophages (81) follicular dendritic cells (82), and intestinal epithelial cells (75).

Both human and mouse CD23 express two isoforms of CD23, which differ by six or seven amino acids in the cytoplasmic tail. These two isoforms, CD23a and CD23b, arise through two transcription initiation sites and alternate splicing of RNA transcripts. How the two isoforms differ is not entirely clear, but binding *via* CD23a appears to induce endocytosis, whereas binding *via* CD23b induces phagocytosis (83).

Surprisingly, the interaction between CD23 and IgE is independent of IgE glycosylation (71, 81), even though IgE binds to the lectin-like head domain of CD23. However, the stalk region might also be directly involved in IgE binding (84). One reason for the glycan-independent binding in humans may be that human CD23 does not bind Ca^2+^ in the classical C-type lectin site, which appears to make it unable to interact with carbohydrates (2, 85). However, the presence of Ca^2+^ may still influence the binding to IgE because Ca^2+^ affects the structure of CD23 and hence enhances the binding (86).

In addition, the valence of the IgE makes a difference. Aggregated IgE or IgE in complex with an antigen and potentially with anti-IgE IgG increases binding to CD23 (3, 28, 87).

While IgE must adopt an open conformation to bind FcεRI, it must be in the closed form for CD23 (88, 89). The formation of IgE-IC results in an increase in the binding of IgE. This suggests that the complexation with the antigen limits the flexibility of IgE, preventing it from adopting the open form. However, it is also possible that complexed IgE binds more effectively to CD23 than monomeric IgE due to increased avidity. Nevertheless, this would not explain the reduced binding to FcεRI.

**Figure 1:**
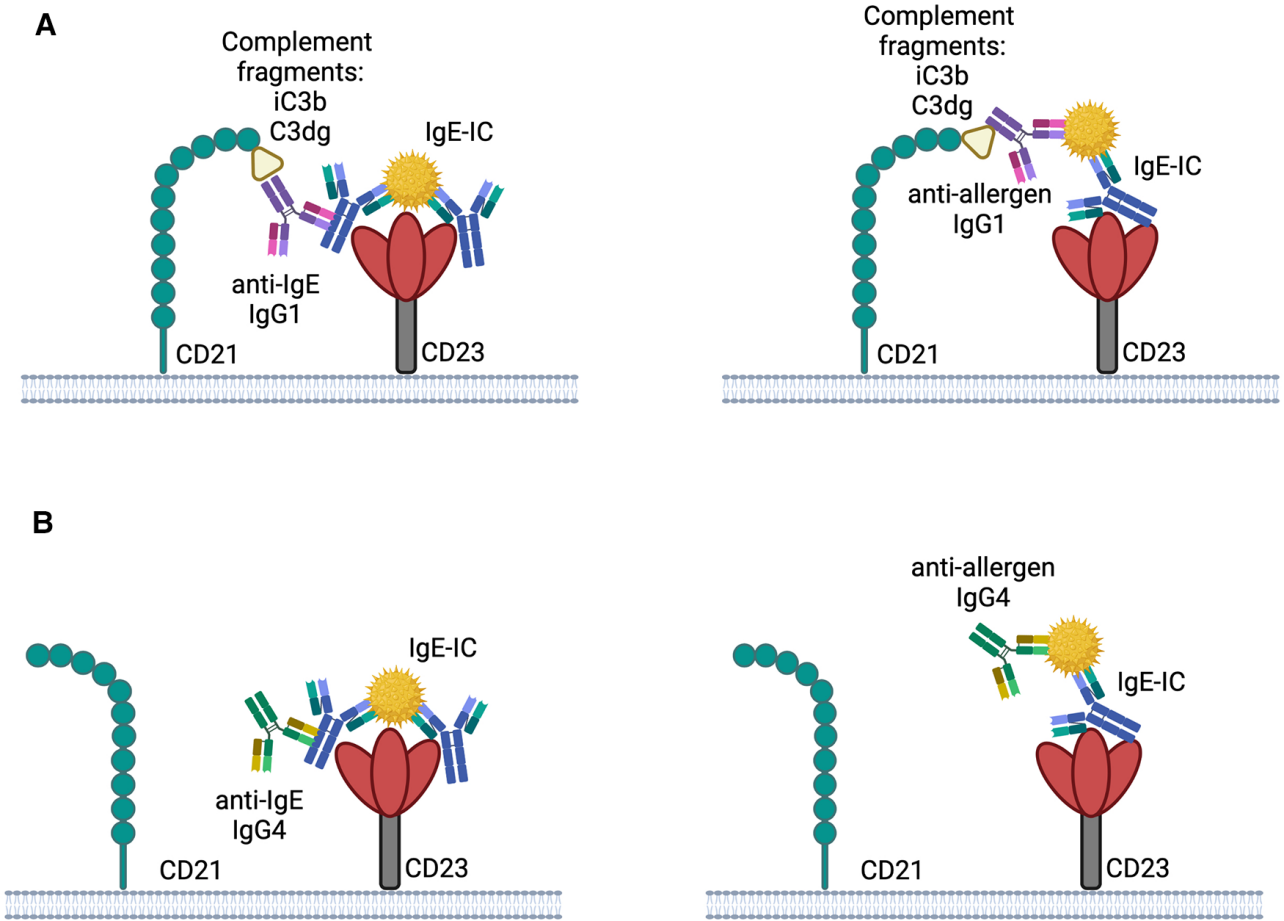
Possible interplay between CD21 and CD23 with IgE-IC the binding of IgE-IC to CD23 may be enhanced by complement fixed anti-IgE IgG1 or anti-allergen IgG1 by simultaneously binding to CD21 (**A**). Binding to CD23 is not improved by CD21 due to the involvement of IgG4 in the IgE-IC, which does not bind complement well (**B**). The illustrations are created with BioRender.com.

The binding of IgE-ICs to CD23 can be influenced by other receptors, such as the complement receptor CD21, which binds to human but not to mouse CD23 (90). While IgE alone doesn't activate the complement system, the presence of complement-fixing IgG in the complex can have an impact on its binding to CD23. Studies have revealed that the immune complexes formed in allergy patients contain IgE, IgG1, and IgG4 (91). Immune complexes which contain IgG1 can activate complement leading to the formation of complement protein fragments (iC3b and C3dg), which both bind to CD21 and thereby promote the fixation of the immune complexes to CD21 (Figure 1A). Thus, the interaction between immune complexes and B cells through CD23 could be affected by the presence of specific IgG subclasses. Since IgG4 does not fix complement efficiently, a higher concentration of IgG4 compared to IgG1 in the complex could reduce the interaction between CD23 and CD21 and therefore hinder the targeting of the immune complex to B cells (Figure 1B) (10).

Like FcεRI, CD23 can also exist in a soluble form. Soluble CD23 (sCD23) molecules are formed by the proteolytic cleavage of the transmembrane form of CD23. These cleavage products are produced by intracellular proteolytic processing, metalloproteases of the ADAM family and the house dust mite allergen Der p1 (90–96). sCD23 is mainly produced by B cells and is influenced by expression levels of transmembrane CD23 and rate of proteolytic cleavage. sCD23 increases the production of IgE through co-ligation of CD21 and mIgE (2, 97) The size of clusters induced by sCD23-mediated cross-linking at the cell surface determines the strength of the IgE-inducing signal. However, not all forms of sCD23 are equally potent at inducing IgE-production (98). Short recombinant sCD23 fragments corresponding to sCD23 monomers generated by Der p1 can form only small complexes, which were even inhibitory for IL-4 induced synthesis of IgE in the experimental settings (66).

sCD23 also promotes proliferation and differentiation of various immune cells and enhances production of inflammatory cytokines by binding to various receptors on monocytes and macrophages (72, 73, 99, 100), activating nitric oxide synthase and inducing production of inflammatory cytokines in humans (79, 101, 102) and mice (103).

## Galectins: the glycan-recognizing receptors

Galectins are a large and evolutionarily conserved group of lectins that bind to beta-galactosides (104). They are primarily located in the cytoplasm but can also be found in the nucleus and secreted into the extracellular environment (105). Galectins play a role in intracellular processes such as mRNA splicing, cell differentiation, and apoptosis (106). Secreted galectins promote intercellular communication, such as cell migration, or contribute to cell adhesion to extracellular matrix proteins, such as laminin and fibronectin (107).

Galectin-3 (Gal-3) has been identified as an IgE-binding receptor that can also interact with FcεRI and activate mast cells (11–13). Gal-3 is expressed by various cell types under normal conditions, such as epithelial cells, dendritic cells, macrophages, and neutrophils (106–109). However, inflammatory responses can modulate expression of Gal-3 (106, 108, 110, 111). Gal-3 has multiple functions including phagocytosis, leukocyte recruitment, and effects on apoptosis (15–18).

The role of Gal-3 in allergic reactions is complex and depends primarily on whether it is located intracellularly or extracellularly. Extracellularly, Gal-3 has been shown to promote allergic inflammation by facilitating the migration of eosinophils to the airways (21, 22) and increasing IgE levels in a mouse model of atopic dermatitis (23). Additionally, Gal-3 has been identified as a potent activator of mast cells, both by cross-linking FcεRI-bound IgE and directly cross-linking FcεRI on basophils. This leads to the release of pro-inflammatory cytokines, such as IL-4 and IL-13, further exacerbating allergic inflammation and promoting TH2 polarization (24).

In contrast to its extracellular effects, intracellular Gal-3 has been shown to negatively regulate mast cell activation in mice. Using Gal-3 knockdown bone marrow-derived mast cells (BMMCL), Bambouskova et al. found that intracellular Gal-3 impairs degranulation and mediator release by decreasing phosphorylation of signaling molecules and calcium response, as well as by promoting internalization of IgE-FcεRI complexes upon antigen triggering (112). Additionally, intracellular Gal-3 appears to play a role in regulating mast cell adhesion, motility, and chemotaxis by stabilizing beta 1 integrin on the cell membrane and promoting proper adhesion to fibronectin (112). It's important to note that the research findings in mice may not reflect the effects of human Gal-3. In fact, a significant number of *in vivo* and *in vitro* studies indicate that Gal-3 is mostly pro-inflammatory (113).

Since the binding of Gal-3 to IgE is glycan-dependent, it is not surprising that differentially glycosylated IgE antibodies show unique Gal-3 binding capabilities (24, 114). However, it remains unclear whether the fact that IgE from healthy and atopic individuals bind differently to Gal-3 is due to potential variations in glycosylation patterns. Interestingly, sialidase-treated IgE has a higher binding affinity to Gal-3 than untreated human IgE on which more terminal galactose residues are displayed (24, 114). Additionally, Shade et al. showed that healthy individuals possess more terminal galactose on their IgE molecules compared to individuals with peanut allergies, who have IgE molecules with more sialic acid (41). This might indicate that Gal-3 may interact more effectively with IgE from healthy individuals. However, the significance of this observation remains unclear, and further research is needed to determine if it represents a form of IgE regulation.

The impact of IgE complexation on its binding to Gal-3 remains uncertain. While complexed IgE retains its glycans, a reduced binding may be attributed to changes in conformation or steric hindrance that prevent binding to Gal-3. For FcεRI, IgE glycosylation is essential for binding, although the sugars are not directly involved in binding (115). This is different for Gal-3, where the sugar is the epitope itself (112, 116). Therefore, more experiments are needed to clarify this question.

Galectin-9 (Gal-9) is also known to bind to IgE (14). It is predominantly expressed in immune cells and the epithelial tissue of the gastrointestinal tract (117–120). Like Gal-3, Gal-9 is primarily localized within the cytoplasm, but can also be secreted and exert biological functions outside the cell (14). Gal-9 also binds CD44, a crucial adhesion molecule for eosinophils and lymphocytes (19, 20). Upon binding to Gal-9, IgE loses the ability to bind its specific allergen. Gal-9 exerts anti-inflammatory effects by inhibiting degranulation and calcium mobilization (14). It is speculated that Gal-9 serves as a negative feedback mechanism as its expression on mast cells increases upon stimulation by IgE and allergens (25). However, it is unknown which glycan structures are recognized by Gal-9, nor is it known if the binding of IgE to Gal-9 depends on the IgE clone. It has been hypothesized that Gal-9 may recognize glycans in the Cε1 region since Gal-9 blocks antigen access to IgE and suppresses cell degranulation (14). Additionally, the binding of Gal-9 to IgE in close proximity to the variable region of the antibody may explain why IgE can no longer bind to the allergen in the presence of Gal-9 (25). However, there is currently a lack of direct experimental evidence supporting this hypothesis. Furthermore, it is not known whether Gal-9 binds exclusively to IgE or if IgE-IC can also be a target, although it seems unlikely that Gal-9 would bind to IgE-IC given that Gal-9-bound IgE no longer binds the allergen.

## The Fc gamma receptors for IgE

In addition to FcεRI and CD23, some FcγRs in mice, such as FcγRII and FcγRIII, have been found to bind IgE-allergen immune complexes (IgE-ICs) with an affinity comparable to that of IgG (26). The association constants for FcγRII is 3.1 × 10^5^ M^−1^ and 4.8 × 10^5^ M^−1^ for FcγRIII (26). Furthermore, the binding of IgE-ICs to FcγRII/III activates mast cells, resulting in the release of serotonin (26).

Another IgE-binding FcγR is FcγRIV, an IgG-binding receptor that is exclusively found in mice. It binds IgG2a and IgG2b with an intermediate affinity of K_A_ 2.9 × 10^7^ M^−1^ and 1.7 × 10^7^ M^−1^, respectively (27). FcγRIV functions as an activating receptor *via* the common FcRγ subunit, similar to other activating FcRs (121). It is reported that FcγRIV is a low-affinity receptor for IgE with a K_A_ of approximately 2.5 × 10^5^ M^−1^. In form of immune complex IgE is capable of displacing IgG2 from the receptor (27). Furthermore, FcγRIV has been found to induce TNF-α secretion in macrophages, similar to human FcεRI (αγγ) (27). This has led to the hypothesis that FcγRIV plays a role in mice similar to that of FcεRI (αγγ) in human (27). It is noteworthy that all IgE-binding Fcγ receptors have been found to induce signaling only to complexed or aggregated IgE but not to monomeric IgE. An explanation for this phenomenon may be the avidity effect, which possibly increases the binding affinity of complexed or aggregated IgE to Fcγ receptors. The avidity effect may be further enhanced by altered conformation of IgE in complexes or aggregates, which may lead to an increased binding to Fcγ receptors. However, there is currently no empirical evidence to support the idea that complexed IgE adopts a distinct conformation that enhances binding to specific Fcγ receptors.

There is also some evidence, that binding of IgE-IC to FcγR is glycan-dependent (28). Therefore, it would be interesting to see if deglycosylation of IgE also affects binding to FcγRII, FcγRIII, and FcγRIV.

In humans there is no evidence suggesting that Fc receptors (FcγR) bind to IgE or IgE-allergen immune complexes (IC). However, it may be postulated that IgE-anti-IgE IgG complexes with or without allergen may bind to FcγR like IgE-omalizumab complexes (122). Additionally, there is some evidence that the neonatal Fc receptor (FcRn) binds to IgE-anti-IgE IgG complexes, being thereby responsible for the presence of IgE antibodies in the fetus and for the increase of allergic risk associated with these complexes (123).

## Regulation of IgE and IgE-immune complexes by IgE receptors

The regulation of IgE half-life in serum depends strongly on whether it is present as IgE or as an IgE-immune complex (IgE-IC). Monomeric IgE can bind to both FcεRI and CD23. Since the affinity of FcεRI for IgE is exceptionally high, IgE binds quickly and remains bound to the receptor for a relatively long time (with a half-life of approximately 3 weeks) (124, 125). In contrast, IgE in a complex with an allergen no longer binds effectively to the high-affinity FcεRI. While the binding of IgE to CD23 is enhanced by complexation (3).

**Figure 2:**
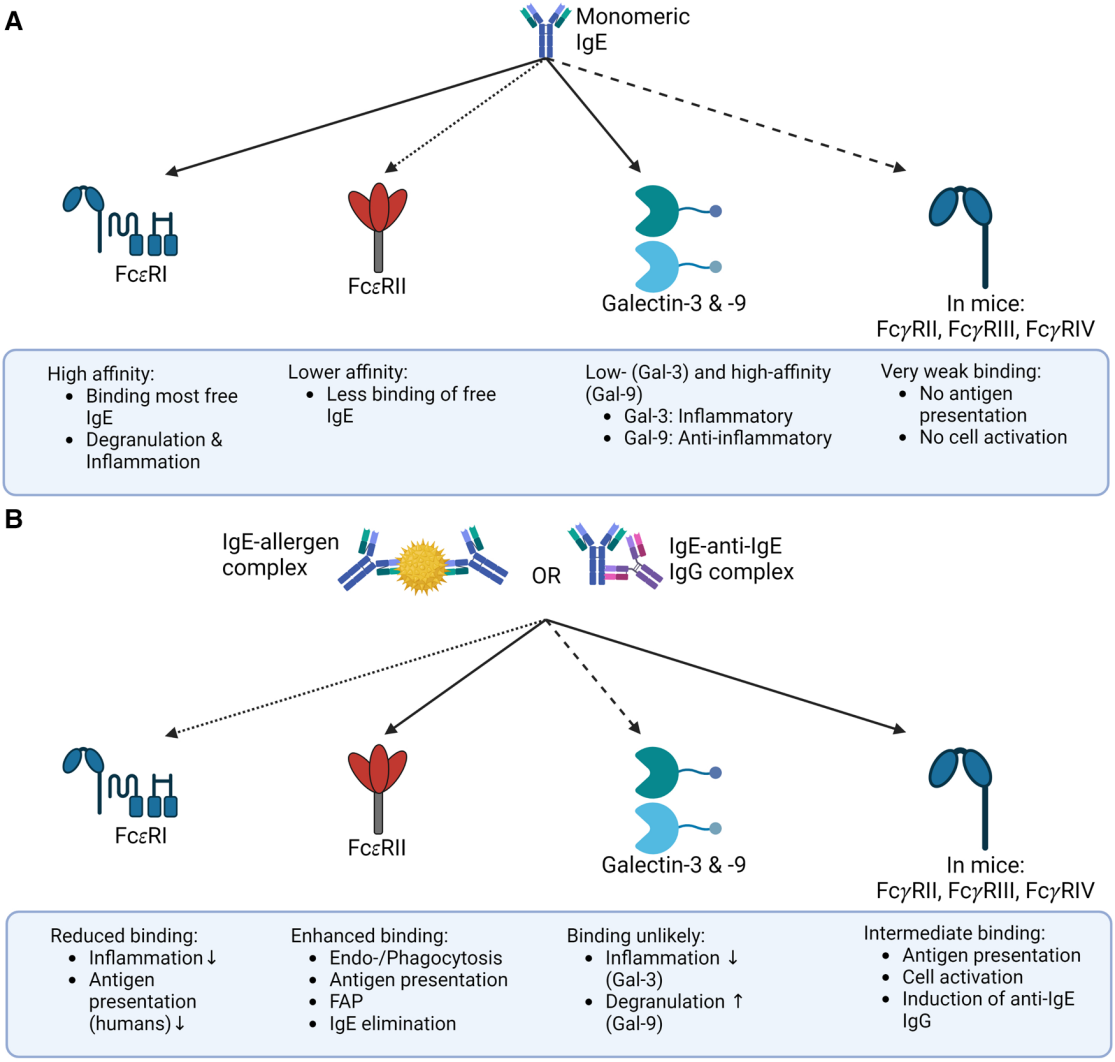
Binding of IgE and IgE-IC to its receptors the binding of IgE to its receptors depends on whether IgE is present as a free monomer or an immune complex. (**A**) Monomeric IgE binds FcεRI, Galectin-3 and -9, and FcεRII (CD23) in mice and humans with different affinities, but only negligible to mouse FcγR. This binding pattern ultimately results in allergic inflammation. (**B**) In contrast, IgE-IC primarily binds to FcεRII in mice and humans and to FcγRs in mice. The binding to FcεRI is significantly decreased, and the ability of Galectin-3 or -9 to bind IgE-ICs is still unclear. This overall results in a non-allergic inflammation primarily due to the reduced binding to FcεRI. The illustrations are created with BioRender.com

As discussed above, the binding of an allergen may influence the conformation of IgE, with an open conformation preferentially binding to FcεRI, and a closed conformation preferentially binding to CD23 (as depicted in Figure 2). This phenomenon could represent a form of negative feedback. Since basophils are relatively short-lived, IgE-IC can prevent the sensitization of new basophils. At the same time, IgE is cleared from the serum by increased binding to CD23 and can no longer sensitize effector cells by binding FcεRI mitigating the risk of systemic anaphylaxis (3).

The fate of IgE-immune complexes (IgE-ICs) after binding to the receptor CD23 is determined by the type of cells that binds the complexes. Interestingly, B cell take up IgE-ICs *via* CD23a, and instead of degrading the complexes, they recycle them back to the cell surface in their native form (7). This allows for the release of antigens over time and the potential for B cells to act as depots for IgE-ICs. We and others have shown that IgE-ICs can be taken up by DCs, which degrade and process them so that they can present peptides *via* MHC class II molecules to T cells (7, 126, 127). However, *in vitro* studies have shown that DCs alone do not induce significant T cell stimulation and that B cells are required for DCs to induce T cell proliferation (7). This CD23-mediated antigen presentation is known as IgE-facilitated antigen presentation (FAP) (8, 9).

It is still not clear how B cells transfer antigens to other cell types. It might be contact-dependent or contact-independent mechanisms such as exosomes (127). Studies have suggested that endocytosed CD23-IgE-IC complexes may reach exosomes on their way to the cell surface. One possible mechanism for this includes the metalloprotease ADAM10 which can bind internalized CD23 resulting in cleavage and sorting into exosomes (96, 128). However, further research is needed to fully understand the mechanism of FAP.

Administration of IgE-IC in mice results in the induction of anti-IgE IgG antibodies (28, 29). Surprisingly, we could also show the presence of these anti-IgE IgG antibodies in FcεRI KO and CD23 KO mice. Therefore, it seems likely that other receptors besides Fcε receptors are involved in binding IgE-ICs (29). Gal-3 and Gal-9, which play a role in basic biological processes such as inflammation, were considered as potential receptors. However, evidence suggests that these receptors are unlikely to bind complexed IgE (as seen in Figure 2). That is why Fcγ receptors seem far more attractive for inducing anti-IgE IgG antibodies (28). In mice, FcγRIIb, FcγRIIIa, and FcγRIV have been shown to bind to IgE-ICs complexes (as seen in Figure 2) (26, 27). It is well established that FcγRI and FcγRIII are able to internalize immune complexes for antigen presentation (129). The role of FcγRIIb in this process is less clear. On B cells, FcγRIIb acts as a regulator of the B cell receptor (BCR), preventing the production of low-affinity and potentially cross-reactive antibodies (130, 131). On the other hand, studies in a mouse model have shown that FcγRIIb can elicit a humoral response upon exposure to IgG immune complexes, although it elicits weaker T cell activation compared to activating FcγRs (132, 133). Given the higher expression levels of FcγRIII and FcγRIV on antigen-presenting cells compared to FcγRIIb, activating FcγRs are more likely targets (134). This hypothesis is supported by the observation that in Fcγ common chain KO mice, the anti-IgE IgG response is reduced following immunization with IgE-ICs, indicating the involvement of FcγRs in this response (28).

The mechanism by which human auto-anti-IgE IgG antibodies are generated remains unresolved, as there is currently no evidence of human FcγRs binding to IgE-IC complexes.

In addition to the mechanisms described above for mice, FcεRI (αγγ) on APCs may also play a role in humans. The role of antigen presentation by the FcεRI in allergy is not clear. Some studies show that antigen uptake by the FcεRI leads to a T_H_2 response and thus may be responsible for initiating allergic reactions (44, 59). In contrast, other studies show that antigen presentation by FcεRI has a positive, allergy-reducing effect (135, 136). However, it is undisputed that APCs can activate T cells by FcεRI. The activated T cells may then activate B cells, leading to the induction of anti-IgE IgG antibodies.

From an immunological point of view, B cells and APCs may handle IgE-ICs differently. B cells are needed to make the antibodies, and APCs are needed to induce T help. As B cells will take up IgE-ICs *via* their IgE-specific B cell receptor, they will automatically process the complexed part (e.g., allergen) for MHC class II presentation. Hence, the IgE-IC receptor for B cells is the B cell receptor specific for IgE, which also mediates uptake and subsequent allergen processing. For APCs, IgE-IC uptake may be facilitated by the receptors mentioned above. However, in allergic individuals, there will be pre-existing allergen-specific help, rendering the role of APCs for priming T help less important. Hence, the role of IgE receptors in driving anti-IgE IgG responses may vary with pre-existing T help.

## Summary

The structure, flexibility, and glycosylation of IgE have a significant impact on its fate. The main inflammatory pathway for IgE, FcεRI, preferentially binds monomeric (3) and glycosylated (30) forms of the antibody. In order to bind to FcεRI, IgE must adopt an open conformation (31, 32). This binding leads to the activation of effector cells and the initiation of inflammation upon encountering the specific allergen. Alternatively, when free IgE binds to an allergen to form an IgE-IC, it binds preferentially to CD23 instead of FcεRI (3), independent of its glycosylation (71, 81). However, for this interaction to occur, IgE must adopt a closed conformation (31, 32). Depending on the isoform of CD23, the IgE-IC can either be degraded (CD23b) (7, 126, 127) or recycled back to the surface, allowing for antigen release over time (CD23a) (7).

Galectins, specifically Gal-3 and Gal-9, also interact with monomeric IgE (11, 12, 14). Gal-3 has been shown to promote allergic inflammation (21, 23, 24), while Gal-9 has been proposed as a negative feedback mechanism, inhibiting degranulation (25). The interaction of IgE and galectins is also dependent on its glycosylation. Further research is needed to understand the potential alterations in binding between differentially glycosylated IgE antibodies in healthy and atopic individuals.

In mice, some FcγRs, such as FcγRII, FcγRIII, and FcγRIV, have been found to bind IgE-ICs, activating mast cells and inducing the production of auto-anti-IgE IgG antibodies (26–28). The role of IgE glycosylation in this process has not been fully explored, but it is thought to be important.

The bigger picture of the interplay between all IgE-binding receptors is still unresolved. More studies are needed analyzing the interplay between IgE, IgE-IC, IgE glycosylation, and their receptors to gain a clearer understanding. A deeper understanding of this will aid in the development of appropriate therapies against IgE-based diseases.
